# A Mixture of U.S. Food and Drug Administration–Approved Monoaminergic Drugs Protects the Retina From Light Damage in Diverse Models of Night Blindness

**DOI:** 10.1167/iovs.19-26560

**Published:** 2019-04

**Authors:** Henri Leinonen, Elliot H. Choi, Anthony Gardella, Vladimir J. Kefalov, Krzysztof Palczewski

**Affiliations:** 1 Gavin Herbert Eye Institute and the Department of Ophthalmology, University of California-Irvine, Irvine, California, United States; 2 Department of Pharmacology, Case Western Reserve University, Cleveland, Ohio, United States; 3 Department of Ophthalmology and Visual Sciences, Case Western Reserve University, Cleveland, Ohio, United States; 4 Department of Ophthalmology and Visual Sciences, Washington University, St. Louis, Missouri, United States

**Keywords:** light damage, night blindness, photoreceptors, therapeutics, vision

## Abstract

**Purpose:**

The purpose of this study was to test the extent of light damage in different models of night blindness and apply these paradigms in testing the therapeutic efficacy of combination therapy by drugs acting on the G_i_, G_s_, and G_q_ protein-coupled receptors.

**Methods:**

Acute bright light exposure was used to test susceptibility to light damage in mice lacking the following crucial phototransduction proteins: rod transducin (GNAT1), cone transducin (GNAT2), visual arrestin 1 (ARR1), and rhodopsin kinase 1 (GRK1). Mice were intraperitoneally injected with either vehicle or drug combination consisting of metoprolol (β_1_-receptor antagonist), bromocriptine (dopamine family-2 receptor agonist) and tamsulosin (α_1_-receptor antagonist) before bright light exposure. Light damage was primarily assessed with optical coherence tomography and inspection of cone population in retinal whole mounts. Retinal inflammation was assessed in a subset of experiments using autofluorescence imaging by scanning laser ophthalmoscopy and by postmortem inspection of microglia and astrocyte activity.

**Results:**

The *Gnat1^−/−^* mice showed slightly increased susceptibility to rod light damage, whereas the *Gnat2^−/−^* mice were very resistant. The *Arr1^−/−^* and *Grk1^−/−^* mice were sensitive for both rod and cone light damage and showed robust retinal inflammation 7 days after bright light exposure. Pretreatment with metoprolol + bromocriptine + tamsulosin rescued the retina in all genetic backgrounds, starting at doses of 0.2 mg/kg metoprolol, 0.02 mg/kg bromocriptine, and 0.01 mg/kg tamsulosin in the *Gnat1^−/−^* mice. The therapeutic drug doses increased in parallel with light-damage severity.

**Conclusions:**

Our results suggest that congenital stationary night blindness and Oguchi disease patients can be at an elevated risk of the toxic effects of bright light. Furthermore, systems pharmacology drug regimens that stimulate G_i_ signaling and attenuate G_s_ and G_q_ signaling present a promising disease-modifying therapy for photoreceptor degenerative diseases.

Photoreceptor degenerative diseases comprise a major cause of vision loss and blindness.^1^ Unlike other forms of major blinding conditions, such as cataract or glaucoma, photoreceptor degenerative diseases unavoidably progress after diagnosis as they lack treatment options. More than 200 genetic causes for photoreceptor degenerative diseases have been found to date (https://sph.uth.edu/retnet/, provided by The University of Texas Health Science Center, Houston, TX, USA). However, the most common photoreceptor degenerative condition, age-related macular degeneration, is multifactorial, often sporadic, and generally cannot be delineated to a single cause.^2^ Therefore, a generic treatment option for these diseases, rather than correction of a single pathological entity, is highly desirable. One generic treatment strategy is neuroprotective therapy intended to combat neurodegenerative brain diseases regardless of their underlying causative mechanisms.^3^ Such a disease-modifying treatment strategy does not intend to correct the primordial cause of disease but, instead, aims at blocking the key pathological pathway(s) that drive cell dysfunction and death.

One recently proposed disease-modifying treatment strategy for retinal degeneration relies on systems pharmacology in which a combination of drugs targets several subtypes of G protein-coupled receptors (GPCRs) simultaneously, specifically the G_s_-, G_i_-, and G_q_-coupled receptors.^4^,^5^ G_s_- and G_i_-coupled receptors both regulate adenylate cyclase. Agonism at the G_s_-coupled receptor increase the activity of adenylate cyclase, leading to increased turnover of cAMP, whereas agonism at the G_i_-coupled receptor has an opposite inhibitory effect. The G_q_-coupled receptor–signaling pathway differs from G_s_- and G_i_-coupled receptors in that it signals via the phospholipase C pathway eventually leading to Ca^2+^ release from the endoplasmic reticulum. However, the G_s_-, G_i_-, and G_q_-coupled receptors' signaling effects converge as intracellular cAMP levels modulate Ca^2+^ levels and vice versa.^6^ This systems pharmacology approach takes into account the cooperation, flexibility, and redundancy of the aforementioned biochemical pathways and decreases the cells' propensity to compensate toward homeostasis if only one of the elements is targeted.^4^ Indeed, it was previously shown that simultaneous administration of a G_i_-receptor agonist (bromocriptine [BRM]) and G_s_- and G_q_-receptor antagonists (metoprolol [MET] and tamsulosin [TAM], respectively) lead to a stronger therapeutic effect than a sum of their parts (i.e., synergistic effect).^7^

The first steps of vision begin with the conversion of photons at visible wavelengths of light into electrical signals in the retina.^8^ Once a photon reaches the retina and is absorbed by rhodopsin in rod photoreceptors, a reaction is triggered in which rhodopsin's chromophore 11-*cis*-retinal is photoisomerized to all-*trans* configuration activating the rhodopsin molecule.^9^ The activated rhodopsin makes repeated contacts with multiple G-protein transducins, catalyzing their activation, which in turn starts a cascade leading to hyperpolarization of the photoreceptor. Fast recovery from light activation requires a rapid turnoff mechanism that is achieved by rhodopsin's partial inactivation by phosphorylation catalyzed by rhodopsin kinases. Once visual arrestin binds to the phosphorylated rhodopsin, the visual pigment is fully inactivated.^10^ Proper functioning of the phototransduction cascade, including its efficient shutoff, is a prerequisite for normal vision and a healthy retina. In this study, we investigated the extent to which genetic knock-outs of rod and cone transducin (*Gnat1* and *Gnat2*, respectively), rhodopsin kinase 1 (*Grk1*), and visual arrestin 1 (*Arr1, SAG*) predispose mice to the damaging effects of light. Furthermore, we simultaneously tested if the previously effective systems pharmacology-based therapy in the Stargardt disease model^7^,^11^,^12^ could also prevent light damage exacerbated by defects in the phototransduction cascade. Herein we demonstrate that a genetic knock-out of *Gnat1*, associated with congenital stationary night blindness,^13^ slightly increases the susceptibility to light damage in mice, and this can be fully prevented by pretreatment with MET+BRM+TAM. Light damage in *Arr1* and *Grk1* knock-out mice, both associated with Oguchi disease,^14^ was extremely robust, but could also be fully prevented with increasing doses of MET+BRM+TAM.

## Materials and Methods

### Animals

All experiments were approved by the Institutional Animal Care and Use Committees at Case Western Reserve University (IACUC Protocol 2014-0071) and University of California Irvine (IACUC Protocol AUP18-124) and were conducted in accordance with the Association for Research in Vision and Ophthalmology Statement for the Use of Animals in Ophthalmic and Visual Research.

Four genetically engineered mouse lines associated with human retinal diseases caused by defective function of phototransduction proteins, namely congenital stationary night blindness (*Gnat1^−/−^*), achromatopsia (*Gnat2^−/−^*), and Oguchi disease (*Grk1^−/−^* and *Arr1^−/−^* mice), were used in this study. All experiments were performed in 5- to 10-week-old male and female mice. Mice were maintained at ad libitum feeding and 12 hour/12 hour light-dark cycle unless otherwise noted. *Gnat1^−/−^* mice were generated on a mixed background.^15^ *Gnat1^−/−^* mice carried a wild-type (WT) *Rpe65* gene (Leucine at residue 450). *Gnat2^−/−^* mice on a C57BL/6J background were a kind gift from Dr. Marie Burns (University of California, Davis, CA, USA).^16^ *Grk1^−/−^* and *Arr1^−/−^* mice were obtained from Washington University (St. Louis, MO, USA).^17^ The *Grk1^−/−^* mice were originally generated in a C57/B6 background,^18^ but were later backcrossed to a 129S1/SvlmJ (https://www.jax.org/strain/002448, provided by The Jackson Laboratory, Bar Harbor, ME, USA) background. The *Arr1^−/−^* mice were on a C57BL/6J background (https://www.jax.org/strain/000664, provided by The Jackson Laboratory). The *Grk1^−/−^*, *Arr1^−/−^*, and *Gnat2^−/−^* mice carried the Met450 variant of RPE65, which is a ‘protective' variant compared to Leu450 variant.^19^,^20^ *Grk1, Arr1, Gnat1,* and *Gnat2* genotypes and Leu versus Met amino acid conversion at residue 450 in the *Rpe65* gene was tested by PCR. WT BALB/cJ albino mice (https://www.jax.org/strain/000651) were used to compare the extent of light damage to *Gnat1^−/−^*, *Grk1^−/−^*, and *Arr1^−/−^* mice as BALB/cJ mice are commonly used in similar experiments.^21^ Table 1 summarizes the background of mouse models used in this study.^15^,^16^,^18^,^22^

**Table 1:** Summary of Mouse Model Genotypes

**Mouse Strain**	**Background**	**Pigment**	***Rpe65*** **Phenotype**	**Original Reference**
*Gnat1* mutant	129S1/SvlmJ	Agouti	Leucine at 450 residue	Calvert et al. 2000^15^
*Gnat2* mutant	C57BL/6J	Black	Methionine at 450 residue	Ronning et al. 2018^16^
*Grk1* mutant	129S1/SvlmJ	Agouti	Methionine at 450 residue	Chen et al. 1999^18^
*Arr1* mutant	C57BL/6J	Black	Methionine at 450 residue	Xu et al. 1997^22^
BALB/cJ wild-type	n.a.	Albino	Leucine at 450 residue	n.a.

n.a., not applicable.

### Bright Light Exposure (BLE) and Drug Therapy

We used acute BLE to induce rapid photoreceptor degeneration in mice. After an overnight dark adaptation, the mice were intraperitoneally (i.p.) injected with either drug solution or vehicle (2% dimethyl sulfoxide, 2% propylene glycol, and 96% saline; 150 μl volume), and their pupils were dilated with 1% tropicamide eye drops under dim red light conditions. The mice were maintained dark adapted, and 0.5 hours after the injection, they were transferred to fresh Plexiglas home cages surrounded by LED flood lights (85–265 V, 100 W, 6500 K color temperature). These LEDs delivered the BLE. Luminance measured at the center of the cage was set at 12.5 klux or 25 klux when the light sensor was positioned upward (L203 Photometer; Macam Photometrics Ltd., Livingston, UK). Freely moving mice (2–4 per exposure cage) were exposed to bright light for 0.5 hours or 1 hour and were transferred back to the vivarium thereafter.

For systems pharmacology–based therapy,^7^ we used a combination of the following three GPCR drugs: MET, BRM, and TAM. MET and TAM were purchased from TCI America (Portland, OR, USA). BRM was purchased from Enzo Life Sciences (Farmingdale, NY, USA). The drugs were always administered half an hour prior to the induction of BLE as a combination of the three drugs in a volume of 150 μl. The number of replicates in each drug experiments are presented in Table 2.

**Table 2:** Number of Replicates in Each Drug Experiment

**Mouse Strain**	**M+B+T Dose (mg/kg), or Vehicle**	BLE* Parameters	***n*** **(OCT/Flat Mount)**	**Data Presented**
*Gnat1^−/−^*	Vehicle	12.5 klux, 0.5 hours	7/7	Figure 3M/N
*Gnat1^−/−^*	0.2-0.02-0.01	12.5 klux, 0.5 hours	6/7	Figure 3M/N
*Gnat1^−/−^*	1-0.1-0.05	12.5 klux, 0.5 hours	6/5	Figure 3M/N
*Gnat1^−/−^*	10-1-0.5	12.5 klux, 0.5 hours	5/4	Figure 3M/N
*Gnat1^−/−^*	Vehicle	25 klux, 1 hour	5/4	Figure 3O/P
*Gnat1^−/−^*	10-1-0.5	25 klux, 1 hour	5/4	Figure 3O/P
*Gnat1^−/−^*	50-5-2.5	25 klux, 1 hour	4/4	Figure 3O/P
*Grk1^−/−^*	Vehicle	12.5 klux, 0.5 hours	6/7	Figure 4A/E
*Grk1^−/−^*	1-0.1-0.05	12.5 klux, 0.5 hours	6/6	Figure 4A/E
*Grk1^−/−^*	10-1-0.5	12.5 klux, 0.5 hours	6/7	Figure 4A/E
*Grk1^−/−^*	50-5-2.5	12.5 klux, 0.5 hours	5/5	Figure 4A/E
*Arr1^−/−^*	Vehicle	12.5 klux, 0.5 hours	8/7	Figure 5A/E
*Arr1^−/−^*	1-0.1-0.05	12.5 klux, 0.5 hours	5/5	Figure 5A/E
*Arr1^−/−^*	10-1-0.5	12.5 klux, 0.5 hours	6/6	Figure 5A/E
*Arr1^−/−^*	30-3-1.5	12.5 klux, 0.5 hours	5/5	Figure 5A/E

ONL thickness is an average of two eyes, whereas cone preservation analysis is from one eye per mouse.

* BLE, bright light exposure at an average exposure intensity of 12.5 or 25 klux, and 0.5- or 1-hour duration.

Several dosages were used in different experiments, ranging 0.2 to 50 mg/kg (body weight [bw]) for MET, 0.02 to 5 mg/kg (bw) for BRM, and 0.01 to 2.5 mg/kg (bw) for TAM. A simple practice guide for dose conversion between animals and humans suggests a human reference weight of 60 kg.^23^ A typical daily dose of MET in the treatment of blood pressure ranges from 25 to 100 mg (https://www.rxlist.com/, provided by WebMD, New York, NY, USA), and assuming a 60-kg person, this equals to ∼0.4 to 1.7 mg/kg. The corresponding calculations for BRM (20–30 mg daily dose for acromegaly) and TAM (0.4–0.8 mg for prostatic hyperplasia) brings us to ∼0.3–0.5 mg/kg and 0.007–0.01 mg/kg, respectively. Thus, the lowest doses we used in this study are at clinical dose level or lower, and in our previous mass spectrometry study,^12^ these doses given by i.p. injection rendered such low plasma concentration in mice that they fell below the detection limit. Rather, 5-fold higher doses at 1 mg/kg MET, 0.1 mg/kg BRM, and 0.05 mg/kg TAM were needed to bring the drug plasma concentrations to ∼0.1-2 ng/ml range, which is rather small when compared with the reported therapeutic blood levels for MET, BRM, and TAM in humans.^24^–^26^

### In Vivo Retina Imaging by Optical Coherence Tomography and Scanning Laser Ophthalmoscopy

All mice underwent optical coherence tomography (OCT) imaging 7 days after BLE. The mice were anesthetized with ketamine (20 mg/ml, KetaVed, Bioniche Teoranta, Inverin Co, Galway, Ireland) and xylazine (1.75 mg/ml, Rompun, Bayer, Shawnee Mission, KS, USA) solution in PBS at a dose of 0.1 to 0.13 ml/25 g (bw) by i.p. injection, and their pupils were dilated with 1% tropicamide. OCT was performed with a Bioptigen spectral-domain OCT device (Leica Microsystems Inc., Buffalo Grove, IL, USA). Four frames of OCT b-scan images were acquired from a series of 1200 a-scans. Retinal outer nuclear layer (ONL) thickness was measured 500 μm away from the optic nerve head (ONH) in four retinal quadrants (nasal, temporal, superior, and inferior) using a ruler tool in ImageJ 1.52a software (National Institutes of Health, Bethesda, MD, USA). For a majority of the analyses, ONL thickness was averaged over the four retinal quadrants, and this average was normalized to the average ONL thickness before the induction of light damage.

Scanning laser ophthalmoscopy (SLO) was performed to obtain whole-fundus images in vivo from a subset of mice right prior to the OCT. We used the Heidelberg Retinal Angiograph II (Franklin, MA, USA) SLO machine in the autofluorescence mode for these images, analyzed qualitatively.

### Eye Sample Preparation and Immunohistochemistry

After in vivo imaging, the mice were euthanized by cervical dislocation. The superior side of the eyes was marked with a permanent marker, and thereafter the eyes were enucleated. One eye was inserted into 4% paraformaldehyde (PFA) in PBS, and the other eye was either fixed similarly in 4% PFA or in Hartman's fixative (Sigma Aldrich, St. Louis, MO, USA). The eyes were kept in Hartman's fixative for 24 hours and subsequently embedded into paraffin and sectioned at 7 μm thickness in nasal-temporal orientation (yields vertical, superior-inferior oriented sections). These sections were stained with hematoxylin and eosin (H&E). The PFA-fixed eyes were kept in PFA for 1 hour after enucleation, and subsequently the retina was dissected away and processed as a whole-mount sample and then stained using polyclonal goat S-opsin (1:1000 dilution, custom-made; Bethyl Laboratories, Montgomery, TX, USA) and polyclonal rabbit M-opsin (1:1000 dilution, cat. NB110-74730; Novus Biologicals, Littleton, CO, USA) primary antibodies and fluorescent secondary antibodies (dilution for both 1:500; donkey anti-goat Alexa Fluor 488, and donkey anti-rabbit Alexa Fluor 647; Abcam, Cambridge, UK), as described previously.^12^ A subset of whole-mount retinas were processed to inspect astrocytes and microglial cells and therefore stained using monoclonal mouse anti-GFAP (1:500 dilution; Cell Signaling Technology, Danvers, MA, USA) and polyclonal rabbit anti-Iba1 (1:500 dilution; Wako Chemicals USA Inc., Richmond, VA, USA) primary antibodies, and fluorescent secondary antibodies (dilution for both 1:500; Abcam donkey anti-goat Alexa Fluor 488, and donkey anti-rabbit Alexa Fluor 594).

### Light and Confocal Microscopy and Cone Photoreceptor Preservation Analysis

H&E-stained coronal eye cross-section images were captured using a light-microscope (Olympus FSX100; Olympus, Waltham, MA, USA). Individual images were captured at 20× magnification and stitched together by the build-in software. Flat-mount retinas were imaged with a fluorescent light-microscope (Leica DMI6000B) equipped with an automated stage. Individual images were captured at 20× magnification, and these images were stitched together using MetaMorph 7.8 software (Molecular Devices, Sunnyvale, CA, USA) to create retinal whole-mount panoramic images by using green (excitation 480/40 nm) or far red (excitation 620/60 nm) fluorescence filter channels. Cone-area preservation after BLE injury was inspected manually from whole-mount panoramic images. The entire retinal area, ONH area, and damaged area were visually determined and manually drawn using MetaMorph software's trace region tool by a researcher blinded as to the experimental parameters. The damaged area was determined for both S-opsin and M-opsin positive images, and the percentage of damaged area compared with the whole retinal area (ONH area subtracted) was used in the statistical analyses. The data are presented as cone preservation percentage compared with baseline. Note that 100% indicates fully preserved and completely healthy retina in terms of cone population.

Confocal microscopy was performed using the Leica TCS SP8 STED microscope, and the images were captured using a 63× oil-immersion objective. A central location of the retina was scanned throughout its width in 1-μm steps using gate reference wavelengths 488 nm and 594 nm. A Z-stack of 10 images (10 μm depth) was combined at the depth of the outer plexiform layer and the retinal nerve fiber layer, representing microglial cells and astrocytes, respectively.

### Statistical Analysis

Statistical analyses were performed using GraphPad Prism 8 software (La Jolla, CA, USA). Two-way repeated measures analysis of variance (ANOVA) with Geisser-Greenhouse correction was applied to datasets that had two variables (retinal location and thickness). One-way ANOVA was applied to the analyses of average ONL thickness. All ANOVAs were followed by Bonferroni post hoc tests. Cone-area preservation data were analyzed by nonparametric Kruskal-Wallis test followed by the Dunn's multiple-comparison tests. Data are presented as means ± SEM. The level of statistical significance was set at *P* < 0.05.

## Results

### Defective Function of Rod Transducin, but not Cone Transducin, Exacerbates Light Damage in Mice

**Figure 1:**
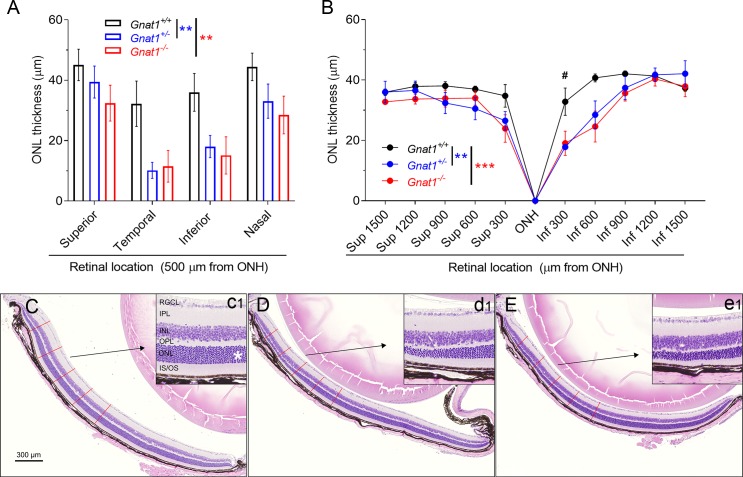
Homozygous and heterozygous knockouts of rod-transducin (Gnat1) slightly increase susceptibility to light damage in mice. (A) ONL (i.e., photoreceptor nuclei layer) thickness analysis from OCT images taken at 7 days after BLE shows that Gnat1^+/−^ (blue bars, n = 8) and Gnat1^−/−^ (red bars, n = 5) have thinned ONL when compared with Gnat1^+/+^ (black bars, n = 7) mice. Geisser-Greenhouse corrected RM ANOVA was used followed by Bonferroni's post hoc tests to compare between-subjects main effect: Gnat1^+/+^ vs. Gnat1^+/−^ and Gnat1^+/+^ vs. Gnat1^−/−^. Significance denoted by **P < 0.01. (B) Morphometry analysis from histological sections shows that central inferior (Inf) retina is thinned both in Gnat1^+/−^ (n = 7) and Gnat1^−/−^ (n = 5) mice in relation to the Gnat1^+/+^ (n = 6) mouse, but superior (Sup) retina is relatively spared. Geisser-Greenhouse corrected RM ANOVA was used followed by Bonferroni's post hoc tests to compare between-subjects main effect: Gnat1^+/+^ vs. Gnat1^+/−^ and Gnat1^+/+^ vs. Gnat1^−/−^. Significance denoted by **P < 0.01 and ***P < 0.001. Post hoc test to compare the genotypes in different locations: ^#^P < 0.05. (C) A representative sample of H&E stained retinal cross-section from a Gnat1^+/+^ mouse. Inferior retina is shown at ONH level in relation to temporal-nasal orientation. Red lines illustrate the measurement points of ONL thickness used for statistical analysis in image B. The inset (c1) shows a magnification image between 600 and 900 μm from ONH, and the asterisk marks the ONL. (D, E) Similar images as C, but from Gnat1^+/−^ (D and inset d1) and Gnat1^−/−^ (E and inset e1) mice. RGCL, retinal ganglion cell layer; IPL, inner plexiform layer; INL, inner nuclear layer; IS, photoreceptor inner segments; OS, photoreceptor outer segments.

To determine if genetic silencing of rod transducin (GNAT1) increases light damage susceptibility in mice, we exposed *Gnat1^+/+^*, *Gnat1^+/−^*, and *Gnat1^−/−^* female mice to BLE at 12.5 klux for 0.5 hours, performed OCT imaging 7 days later, and subsequently euthanized the mice and collected the eyes for histology. Coronal eye cross-sections were prepared for morphometry analysis. ONL thickness was measured from both the OCT images and H&E-stained sections. OCT image analysis revealed that both the heterozygous *Gnat1^+/−^* and homozygous *Gnat1^−/−^* mice were slightly more susceptible to the damaging effect of light than their WT littermates (Fig. 1A; *P* < 0.05). Especially damaged quadrants of the retina were the inferior and temporal sides (Fig. 1A). The histological cross-sections allowed an inspection at higher resolution and a comparison of the central and peripheral retina. This analysis further demonstrated that both *Gnat1^+/−^* and *Gnat1^−/−^* were more prone to light damage than *Gnat1^+/+^* mice (Fig. 1B; interaction: *P* < 0.05). Intriguingly, the *Gnat1^+/−^* and *Gnat1^−/−^* mice showed comparable susceptibility to light damage because there was no difference between these groups in either the OCT (*P* = 0.61) or histology image analyses (*P* = 0.65) of the ONL thickness. Notable is that the light damage with these experimental settings was modest and restricted to the central part of the retina, leaving the peripheral retina intact (for illustrative examples, see Figs. 1C–E).

**Figure 2:**
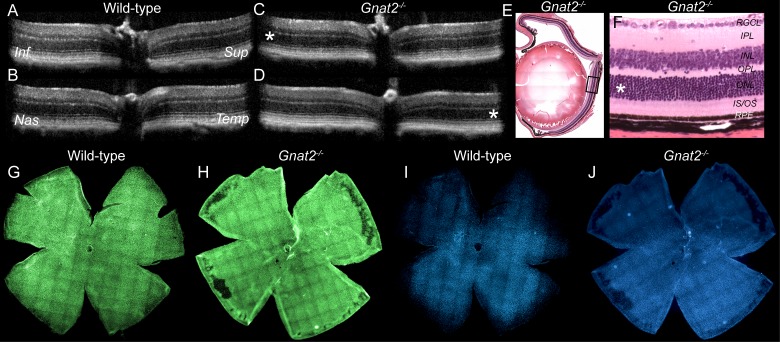
Retinal anatomy analysis by three different methods illustrates resistance to light damage in Gnat2^−/−^ mouse. Imaging was performed 1 week after intense BLE at 25 klux for 1 hour BLE. (A, B) OCT images in superior-inferior (A) and temporal-nasal (B) orientations in a wild-type mouse. (C, D) OCT images in superior-inferior (C) and temporal-nasal (D) orientations in a Gnat2^−/−^ mouse. Asterisks highlight the ONL in the inferior (C) and temporal (D) locations where light damage is typically first observed in pigmented mice. In BLE Gnat2^−/−^ mice, these locations show normal retina morphology. (E) A representative sample of H&E-stained Gnat2^−/−^ mouse eye histology. Black quadrangle illustrates the point at which high magnification image (F) was taken. (F) High-magnification histology image shows completely normal retinal anatomy at the central inferior retina. (G, H) Flat-mounted wild-type (G, I) and Gnat2^−/−^ (H, J) mouse retinas stained against anti M- (green) and S-opsin (blue) antibodies indicate preserved cone photoreceptor population both in wild-type and Gnat2^−/−^ mice. Three replicates showed the same result.

We initially did similar experiments as presented previously using *Gnat2^−/−^* mice that lack the cone transducin and therefore cone-mediated phototransduction,^16^ but found no effect of BLE (data not shown). Therefore, we doubled the light intensity to 25 klux and increased the duration of light exposure to 1 hour. Nevertheless, we did not detect any light damage 7 days after strong BLE in *Gnat2^−/−^* mice as investigated by OCT (Figs. 2C, 2D), histology (Figs. 2E, 2F), or by cone-population analyses (Figs. 2H, 2J).

### Low-Dose MET+BRM+TAM Combination Therapy Alleviates Light Damage in Gnat1^−/−^ Mice

**Figure 3:**
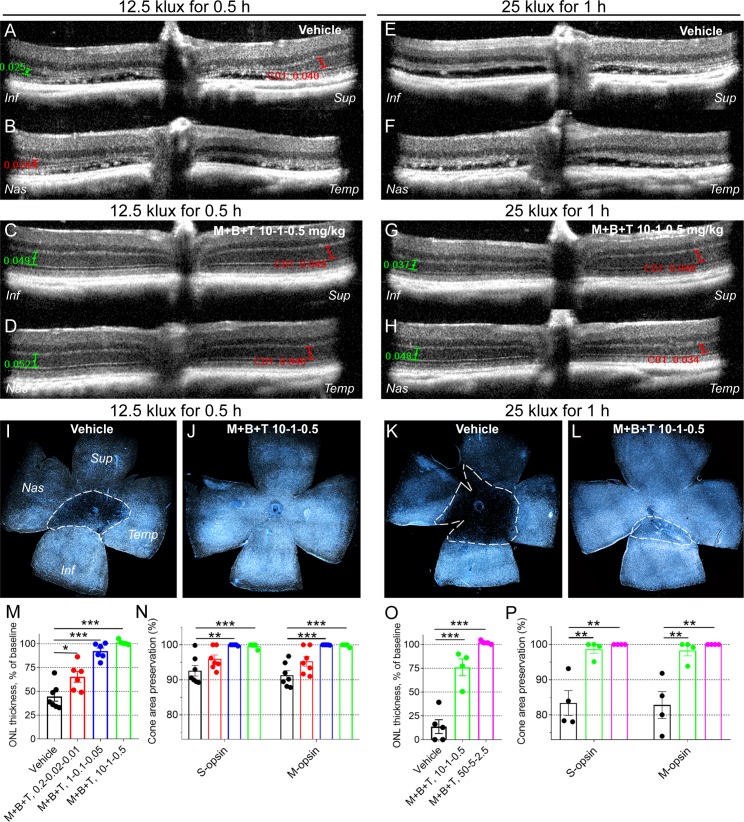
Protection of rods and cones against light damage in Gnat1^−/−^ mice. Mice were subjected to BLE (12.5 klux for 0.5 hours, or 25 klux for 1 hour) 1 week prior to imaging. Either drug combination (metoprolol + bromocriptine + tamsulosin [M+B+T]) or vehicle was injected intraperitoneally 0.5 hours prior to induction of light damage. Number of replicates are presented in Table 2. (A–D) Representative OCT images from vehicle-treated (A, B) and drug-treated (C, D) mice that were exposed to BLE at 12.5 klux for 0.5 hours. ONL thickness was measured at all retinal quadrants at 500 μm distance (green and red capped lines) from ONH, and averaged and normalized to baseline (ONL thickness without BLE) for analysis presented in M and O. (E–H) Representative OCT images from vehicle-treated (E, F) and drug-treated (G, H) mice that were subjected to BLE at 25 klux for 1 hour. (I–L) Representative flat-mount retina images show typical S-cone distribution in vehicle-treated (I, K) and drug-treated (J, L) mouse retinas 1 week after BLE at 12.5 klux for 0.5 h (I, J), and more intense BLE at 25 klux for 1 hour (K, L). Dashed white line illustrates the border between damaged and healthy site in cone population. All flat-mounted retinas were oriented the same way. (M) Statistical analysis of averaged and normalized (to baseline, 52.0 ± 1.0 μm) ONL thickness in vehicle-treated and drug-treated mice subjected to BLE at 12.5 klux for 0.5 hours. (N) Cone population preservation in flat-mount retinas from mice subjected to BLE at 12.5 klux for 0.5 hours. (O) Statistical analysis of averaged and normalized ONL thickness (to baseline 52.0 ± 1.0 μm) in mice subjected to BLE at 25 klux for 1 hour. (P) Cone population preservation in flat-mount retinas from mice subjected to BLE at 25 klux for 1 hour. Note that the retinal damage is more severe after 25 klux than 12.5 klux BLE, and a larger dose of drugs is needed to obtain full retinal protection. Statistical analysis was performed by one-way ANOVA, followed by Bonferroni's post hoc test. Significance denoted by *P < 0.05, **P < 0.01, ***P < 0.001. Inf, inferior; Sup, superior; Nas, nasal, Temp, temporal side of the retina.

We next investigated if a promising retinoprotective therapy could alleviate or block light damage in *Gnat1^−/−^* mice. The mice were subjected to BLE using the following two different exposure parameters: (1) 12.5 klux of 0.5 hours and (2) 25 klux for 1 hour. The MET+BRM+TAM combination was administered 0.5 hours before the BLE, OCT imaging was performed 7 days after, and the mice were euthanized and the eyes collected thereafter. In this dataset, the retinas were processed as flat mounts and stained against S- and M-opsins to investigate cone survival. We found that a very small dose of MET+BRM+TAM at 0.2-0.02-0.01 mg/kg, respectively, provided a significant ∼25% protection against ONL thinning as measured from OCT images (*P* < 0.05; Fig. 3M). The preserved cone area also tended to be higher in MET+BRM+TAM–treated (0.2-0.02-0.01 mg/kg) compared with vehicle-treated mice (Fig. 3N), although this projection was not statistically significant. However, when the drug doses were increased 5-fold to 1-0.1-0.05 mg/kg, full cone preservation was obtained (*P* < 0.001; Fig. 3N), and we observed nearly full protection against ONL thinning (*P* < 0.001; Fig. 3M). A dose of 10-1-0.5 mg/kg MET+BRM+TAM fully protected the *Gnat1^−/−^* mouse retina from BLE at 12.5 klux for 0.5 hours (*P* < 0.001, Figs. 3M, 3N).

**Figure 4:**
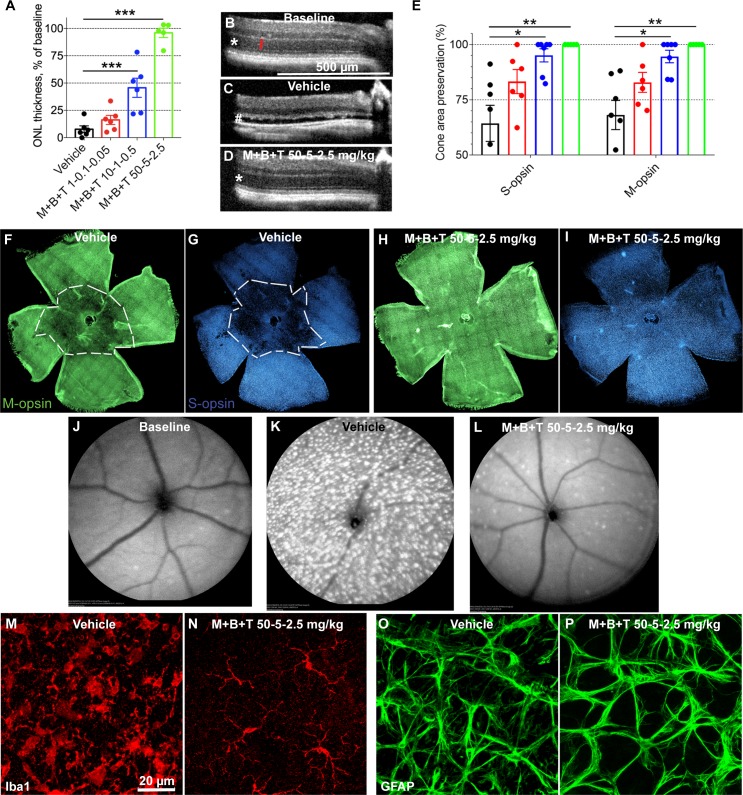
Protection of the retina against light damage in Grk1^−/−^ mice. Mice were subjected to BLE (12.5 klux for 0.5 hours) 1 week prior to imaging. Drug combination (M+B+T) or vehicle was injected i.p. 0.5 hours prior to induction of light damage. Number of replicates are presented in Table 2. (A) Averaged and normalized (to baseline, 58.0 ± 0.2 μm) ONL thickness as measured at all central retinal quadrants (500 μm from ONH) from OCT images. (B–D) Representative OCT images at baseline (B, before BLE) and 7 days after BLE in vehicle (C) and (D) M+B+T–treated Grk1^−/−^ mouse eyes. Asterisks (protection in B, D) and pound sign (damage in C) highlight the region of interest, that is, ONL. Red line in B represents the ONL thickness at 500 μm from the ONH. (E) Damaged cone area in S- and M-opsin labeled flat-mount retinas was manually determined (see Materials & Methods for details). (F–I) Representative flat-mount retina images show typical M- (green) and S-cone (blue) populations in vehicle-treated (F, G) and drug-treated (H, I) mouse retinas 1 week after BLE. Dashed white lines illustrate the border between damaged and healthy site in cone population. Note that M+B+T at a dose 10-1-0.5 mg/kg was able to maintain cone population by ∼95%, whereas ONL thickness (representative of rod population) with this dose was decreased by ∼50%. M+B+T 50-5-2.5 mg/kg dose led to full protection in both rod and cone population. (J–L) Representative autofluorescence fundoscopy images before BLE (J) and 7 days after BLE in vehicle-treated (K) and M+B+T–treated (L) Grk1^−/−^ mouse eyes. (M, N) Representative retinal whole mounts showing microglia (anti-Iba1 staining) at the level of the outer plexiform layer. Note the massive microglial infiltration and amoeboid formation in vehicle-treated mouse retina, whereas the M+B+T–treated mouse retina shows more stratified microglial morphology. (O, P) Representative retinal whole mounts shows slight astrocyte (anti-GFAP staining) activation in vehicle-treated mouse retina, but not in M+B+T–treated mouse retina. Statistical analysis was performed by one-way ANOVA, followed by Bonferroni's post hoc test. Significance denoted by *P < 0.05, **P < 0.01, ***P < 0.001.

We continued by increasing the BLE parameters to 25 klux for 1-hour exposure to test drug efficacy against more severe light damage in *Gnat1^−/−^* mice. Indeed, retina flat-mount images clearly illustrate a larger damage area in a vehicle-treated *Gnat1^−/−^* mouse retina exposed to 25 klux for 1 hour when compared with a vehicle-treated *Gnat1^−/−^* mouse retina exposed to 12.5 klux for 0.5-hour BLE (Figs. 3I, 3K). The same direction can be observed by comparing ONL thicknesses (Figs. 4A, 4B vs. 4E, 4F & Figs. 4M, 4O). Nevertheless, a dose of 10-1-0.5 mg/kg MET+BRM+TAM provided full cone protection in 3 of 4 analyzed eyes even at the more intense BLE at 25 klux for 1 hour (*P* < 0.01; Fig. 3P). The only retina from a MET+BRM+TAM–treated mouse that had some damage is illustrated (Fig. 3L) and shows a significantly smaller damage area than the retina from a vehicle-treated mouse (Fig. 3K). There was small damage to an ONL layer so that 25% of its thickness on average was lost despite treatment with 10-1-0.5 mg/kg MET+BRM+TAM (Fig. 3O). However, when we increased the MET+BRM+TAM dose to 50-5-2.5 mg/kg, complete protection to ONL thickness and cone population was achieved (*P* < 0.01; Figs. 3O, 3P).

### Dysfunctional Rhodopsin Inactivation Caused by Either Lack of Rhodopsin Kinase or Visual Arrestin Renders Mice Extremely Sensitive to Light Damage That Is Alleviated by MET+BRM+TAM Treatment

To investigate if phototransduction overdrive, rather than phototransduction blockade (as presented in the previous *Gnat1^−/−^* data), increases light-damage susceptibility, we continued the BLE experiments using *Grk1^−/−^* and *Arr1^−/−^* mice that lack the rhodopsin kinase and visual arrestin 1, respectively. These mutations lead to dramatically prolonged rhodopsin inactivation.^18^,^22^ In conjunction with light-damage susceptibility per se, we tested if MET+BRM+TAM treatment is effective in these paradigms as well. OCT imaging revealed an almost complete destruction of the central retina's ONL in vehicle-treated *Grk1^−/−^* mice (Figs. 4A, 4C), leaving only a few rows of nuclei occasionally into the superior and/or nasal central retina (data not shown). Although the cones are relatively resistant to light damage, the damaged cone area in flat-mounted retinas in vehicle-treated *Grk1^−/−^* mice was on average ∼40%. Notably, the variation in cone-damage area after bright-light exposure is relatively large, and some mice lost most of their cones (see Fig. S1 for illustration). The light damage in the *Grk1^−/−^* mice was so robust that a modest dose of MET+BRM+TAM at 1-0.1-0.05 mg/kg could not provide a statistically significant therapeutic effect, although some tendency for protection could be observed (Figs. 4A, 4E). Instead, a higher dose of 10-1-0.5 mg/kg protected 50% of the ONL thickness (*P* < 0.001; Fig. 4A) and ∼95% of the cone area in retinal flat-mount samples (*P* < 0.05; Fig. 4E), a result that indicates that complete cone protection can be achieved with lower drug doses than that required to retain the full ONL thickness. Increasing the drug doses to 50-5-2.5 mg/kg fully protected most retinas from ONL thinning and cone damage (*P* < 0.01; Figs. 4A, 4D, 4E, 4H, 4I), and completely blocked the retinal inflammation caused by BLE in the *Grk1^−/−^* mice (Figs. 4J–P).

**Figure 5:**
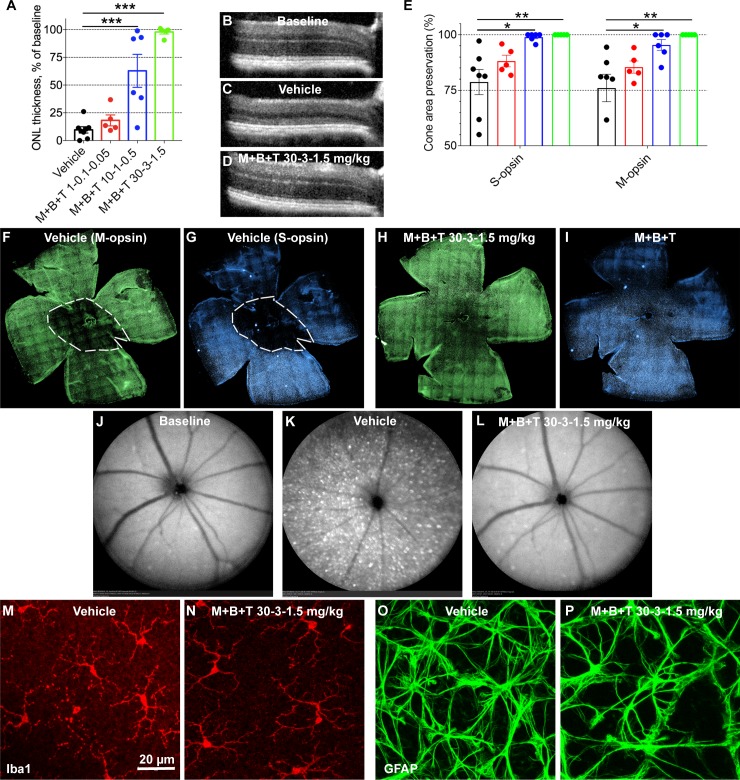
Protection of the retina against light damage in Arr1^−/−^ mice. The design of layout is the same as in Figure 4. Number of replicates are presented in Table 2. (A) Averaged and normalized (to baseline, 59.4 ± 0.3 μm) ONL thickness at central retina. (B–D) Representative OCT images at baseline (B, before BLE) and 7 days after BLE in vehicle-treated (C) and (D) M+B+T–treated Arr1^−/−^ mouse eyes. (E) Damage area in S- and M-opsin labeled flat-mount retinas. (F–I) Representative flat-mount retina images show typical M- (green) and S-cone (blue) distributions in vehicle-treated (F, G) and drug-treated (H, I) mouse retinas 1 week after BLE. Dashed white line illustrates the border between damaged and healthy site in cone population. (J, L) Representative autofluorescence fundoscopy images before BLE (J) and 7 days after BLE in vehicle-treated (K) and M+B+T–treated (L) Arr1^−/−^ mouse eyes. (M, N) Representative retinal whole mounts showing microglia (anti-Iba1 staining) at the level of the outer plexiform layer. Note the amoeboid microglia morphology in the vehicle-treated mouse retina and normal stratified morphology in the M+B+T–treated retina. Note also that the microglia activation was milder in BLE Arr1^−/−^ compared to Grk1^−/−^ mice (compare to Fig. 4M). (O, P) Representative retinal whole mounts show a modest astrocyte (anti-GFAP staining) activation in the vehicle-treated mouse retina, but not in M+B+T–treated mouse retina. Statistical analysis was performed by one-way ANOVA followed by Bonferroni's post hoc test. Significance denoted by *P < 0.05, **P < 0.01, ***P < 0.001.

Similar results were obtained in the *Arr1^−/−^* as with *Grk1^−/−^* mice, with the only difference that light damage seemed slightly milder in the *Arr1^−/−^* mice (inflammatory response comparison in *Arr1^−/−^* and *Grk1^−/−^* in Figs. 4K, 4M, 4O and Figs. 5K, 5M, 5O). Again, a modest dose of MET+BRM+TAM at 1-0.1-0.05 mg/kg could not provide significant retinal protection against light damage in the *Arr1^−/−^* mice, although on average the ONL thickness and preserved cone area were larger in the drug-treated than vehicle-treated mice (Figs. 5A, 5E). A 10-fold higher dose at 10-1-0.5 mg/kg protected more than 50% of the ONL thickness (*P* < 0.001; Fig. 5A) and more than 95% of the cone area (*P* < 0.05, Fig. 5E). A dose of 30-3-1.5 mg/kg practically fully protected the retinas from ONL thinning and cone damage (*P* < 0.01; Figs. 5A, 5D, 5E, 5H, 5I). The MET+BRM+TAM treatment was able to block the BLE-induced inflammation also in the *Arr1^−/−^* mice (Figs. 5J–P).

### Summary of Light-Damage Susceptibility

**Figure 6:**
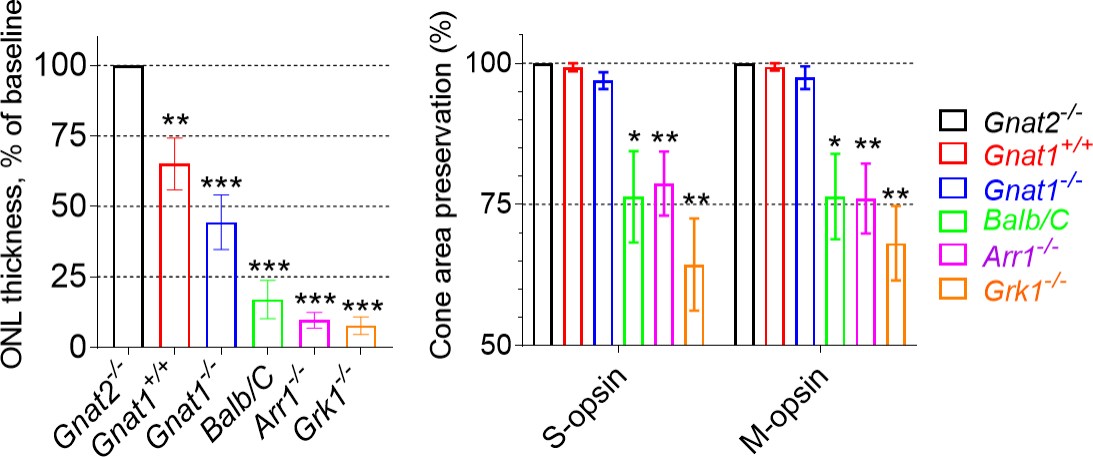
Summary of light-damage susceptibility in mouse strains used in this study. All mice were exposed to BLE at 12.5 klux light for 0.5 hours, and ONL thickness was determined from OCT images 7 days after BLE (Gnat2^−/−^, n = 3; Gnat1^+/+^, n = 13; Gnat1^−/−^, n = 8; BALB/c, n = 8; Arr1^−/−^, n = 8; Grk1^−/−^, n = 6). The retinas were processed as flat mounts 8 days from BLE and stained against opsin antibodies. Cone-area preservation was evaluated from panoramic retina images (Gnat2^−/−^, n = 3; Gnat1^+/+^, n = 7; Gnat1^−/−^, n = 4; BALB/c, n = 9; Arr1^−/−^, n = 7; Grk1^−/−^, n = 7). One-sample t-test was used to test if ONL thickness or preserved cone area was significantly different from 100% (baseline). Significance denoted by *P < 0.05, **P < 0.01, *P < 0.001.

A summary of light-damage susceptibility in the mouse strains used in this study is shown in Figure 6. A group of WT BALB/cJ albino mice was added to this analysis to allow the comparison to this mouse strain commonly used in light-damage studies. The analysis shows that *Gnat2^−/−^* mice (black-colored and carrying methionine mutation at residue 450 in *Rpe65*) were completely resistant to light damage, whereas all other mouse genotypes showed some susceptibility to light damage, including *Gnat1^+/+^* mice that were agouti-colored and WT in terms of the *Rpe65* gene (leucine at residue 450 in *Rpe65)*. When compared with the well-known light-damage susceptible BALB/cJ albino mice (*Leu450*-*Rpe65* gene), our *Arr1^−/−^* (black-colored) and *Grk1^−/−^* (agouti-colored) mice (both carrying *Met450-Rpe65*) showed on average higher, although statistically comparable, susceptibility.

## Discussion

*GNAT1* mutations are causative for autosomal dominant or recessive congenital stationary night blindness. However, two recent clinical reports have implicated the novel type of homozygous *GNAT1* mutations into a progressive retinal dystrophy.^27^,^28^ In *Gnat1^−/−^* mice, a few rows of photoreceptor nuclei are lost within the first weeks of a mouse's life for an yet undetermined reason, but thereafter the condition is stationary.^15^ Using heterozygous and homozygous *Gnat1* knock-out mice, we found that the defective rod phototransduction increases the retina's susceptibility to the damaging effects of light. This was observed as exacerbated thinning of photoreceptor nuclei layer in *Gnat^+/−^* and *Gnat1^−/−^* mice using two different imaging modalities: OCT and light microscopy from eye sections. BLE is commonly used in preclinical research settings to induce photoreceptor degeneration in susceptible albino rodents.^21^,^29^ On the other hand, BLE can be used to trigger photoreceptor degeneration in a timely manner in genetically engineered mouse models of retinal degenerative diseases.^29^ Two previously published works suggest increased susceptibility to light damage in *Gnat1^−/−^* mice.^30^,^31^ Krishnan et al.^31^ showed that the expression of a small number of genes, mostly crystallins, as well as apoptosis were increased in *Gnat1^−/−^* mice retinas. Hao and colleagues^30^ suggested two distinct pathways for light-induced apoptosis in the retina. The first is BLE-induced apoptosis that is independent of transducin. Instead, it is mediated upstream by activated rhodopsin and the subsequent accumulation of toxic visual cycle byproducts, such as all-*trans*-retinal.^30^,^32^,^33^ The second apoptotic pathway appears at low light levels and seems to be primarily dependent on transducin as *Gnat1* deletion blocks low light-induced photoreceptor death in *Grk1^−/−^* mice by 90%.^30^ As BLE-induced damage appears to be mostly mediated upstream of transducin, what then might explain the increased susceptibility in *Gnat1^−/−^* mice remains unanswered. Recently, it was demonstrated that rod phototransduction plays a significant role in adjusting retinal metabolism to changing light conditions.^34^ Researchers showed that retinas extracted from *Gnat1^−/−^* mice had a dysfunction in inhibiting O_2_ consumption and energy metabolism that naturally occur when ambient conditions change from dark to light. This suggests that the retina relies on sensory signaling to match energy production to demand rather than the direct coupling of the two. During BLE, the retina and adjacent retinal pigment epithelium are bombarded with intermediates from the visual cycle such as all-*trans*-retinal. If clearance of all-*trans*-retinal does not match production, toxicity occurs via oxidative stress.^35^ Therefore, the first survival strategy for the retina would be visual cycle shutoff. Indeed, both light and circadian daytime have been shown to partially suppress the rod visual cycle, which likely provides protection from light damage during day time.^36^ Second, metabolic suppression and a decrease in oxidative stress are protective for the retina,^37^,^38^ even if direct evidence from light-toxicity experiments is missing. In the absence of normal rod phototransduction, the metabolic suppression at dark-light switch is dysfunctional,^34^ providing one hypothesis for the subtly increased susceptibility to light damage in *Gnat1* knock-out mice. However, we were surprised that even the heterozygous *Gnat1* knock-out mice showed increased susceptibility to light damage, a finding that to the best of our knowledge has not been shown previously. In the original characterization of *Gnat1* knock-out mice, Calvert et al.^15^ showed that *Gnat^+/−^* mice had very similar phototransduction when compared with WT mice, although the mean sensitivity tended to be lower and displayed an unusually high variability. Their immunoblotting analysis indicated that the α-subunit of transducin, which was the target of knock-out, was decreased only by ∼30% in *Gnat^+/−^* retinas, whereas 100% loss was found *Gnat^−/−^* retinas. Instead, a compensatory increase in expression of β-subunit of phosphodiesterase was equal in *Gnat1^+/−^* and *Gnat^−/−^* retinas. These data imply that the mechanism of increased light-damage susceptibility in *Gnat1^+/−^* and *Gnat1^−/−^* mice can be very complex.

In our study, a lack of phototransduction in the cone pathway did not increase sensitivity to light damage as investigated by using *Gnat2^−/−^* mice. Because only 3% of photoreceptors in mouse retina are composed of cones and the rest are rods, it is conceivable that cone phototransduction has a negligible effect on metabolic shift in the retina. However, our experiment in *Gnat2^−/−^* mice was not completely conclusive as these mice were on the C57BL/6J background, and we could not induce any light damage in either C57BL/6J (data not shown) or *Gnat2^−/−^* mice (both carrying the protective Met450 mutation in *Rpe65*^20^) even at intense light exposure at 25 klux for 1 hour. Other researchers have not found any signs of spontaneous rod or cone degeneration in *Gnat2^−/−^* mice, although full suppression of cone phototransduction in these mice is present.^16^ In humans, *GNAT2* mutations are rare conditions that can cause achromatopsia and/or cone-rod dystrophy.^39^,^40^

Regardless of the cause of increased light damage in *Gnat1* knock-out mice, we were able to prevent it by administering a combination of MET, BRM, and TAM at a relatively low dose pretreatment before BLE. In a report by Chen et al.,^7^ synergistic therapeutic effect against photoreceptor death was achieved when MET, BRM, and TAM were administered simultaneously. The simultaneous administration of several GPCR drugs that inhibit second-messenger signaling decreases the cells' propensity for homeostatic compensation compared to the situation when only one class of GPCRs is targeted.^4^ This enables a stronger therapeutic effect with smaller doses and thus decreased risk of adverse effects. MET is a selective β1-receptor blocker and widely used in the treatment of cardiovascular conditions such as high blood pressure. BRM is mainly used for its agonistic actions on the dopamine family 2 receptors (i.e., D2, D3, and D4) and is also clinically available. TAM is a selective α1-receptor antagonist widely used to treat benign prostatic hyperplasia. Previous research has shown that all of these drugs can prevent light damage in *Abca4/Rdh8* double knock-out mice when given as a high-dose monotherapy.^7^ Although the protective mechanism has not yet been systemically established, it is proposed that decreased second-messenger signaling accounts for this.^4^,^35^ Antagonism at the G_s_-coupled receptor (MET's action) and agonism at the G_i_-coupled receptor (BRM) both decrease the formation of cAMP from ATP via inhibition of adenylate cyclase. TAM inhibits G_q_-coupled receptor mediated signaling and leads to decreased Ca^2+^ release from the endoplasmic reticulum to cytosol via the phospholipase C/inositol triphosphate pathway. Elevated intracellular levels of coregulated second messengers such as Ca^2+^ and cyclic nucleotides can all be cytotoxic by multiple mechanisms.^41^–^43^ Our present data show that low doses of 0.2 mg/kg MET, 0.02 mg/kg BRM, and 0.01 mg/kg TAM partially protected *Gnat1^−/−^* mouse retinas from light damage. Even if direct translation of dosages from mouse experiments to clinical practice is troublesome, we can speculate that these doses are in similar range, or lower, than is generally used with MET, BRM, and TAM for their primary clinical indications (see the “Bright Light Exposure (BLE) and Drug Therapy” section).

Predisposition to light damage in *Grk1^−/−^* and *Arr1^−/−^* mice is known from previous literature.^18^,^22^,^30^,^31^ The mechanism leading to this can be directly delineated to dramatically prolonged activation of rhodopsin. The visual disorder associated with both *GRK1* and *ARR1* mutations is called Oguchi disease and is clinically characterized by a golden-yellow discoloration of the fundus that disappears after prolonged dark adaptation.^44^ Oguchi disease is mostly considered a stationary condition; however, retinal degenerative changes, sometimes severe, have been observed in patients affected with various types of *GRK1* or *ARR1* mutations.^45^–^47^ In the current study, the severity of light damage in *Grk1^−/−^* and *Arr1^−/−^* was still surprising to us, especially considering that our *Grk1^−/−^* and *Arr1^−/−^* mice carried the Met450 conversion in *Rpe65* gene, a mutation that is known to decrease light damage significantly.^20^ We compared the light damage in *Grk1^−/−^* and *Arr1^−/−^* mice to Balb/C albino mice (carrying Leu450 isoform of *Rpe65*) frequently referenced in publications.^21^ The same BLE method caused damage at a similar range in the *Grk1^−/−^* and *Arr1^−/−^* mice when compared with the Balb/C mice, although the *Grk1^−/−^* and *Arr1^−/−^* mice should be relatively protected because of their pigmentation and protective Met450 variant of *Rpe65*. The *Grk1^−/−^* mice displayed massive microglia infiltration and activation 7 days after BLE as shown by intense fundus autofluorescence by SLO imaging and by postmortem immunohistochemistry analyses. A parallel but slightly milder finding was observable in the *Arr1^−/−^* mouse retinas after BLE. The fact that our *Grk1^−/−^* mice were agouti-colored and *Arr1^−/−^* mice were black can explain this difference because the amount of pigment is known to positively correlate with protection of light damage.^48^ Despite the dramatic light damage in the *Grk1^−/−^* and *Arr1^−/−^* mice, we could prevent the associated cone death almost fully with a dose of 10 mg/kg MET, 1 mg/kg BRM, and 0.5 mg/kg TAM. Notable is that the same dose protected only ∼50% of ONL thickness, which is more representative of rod faith.^12^ In light damage and in most genetic causes of photoreceptor degeneration, the rods are primarily dying followed by cone death.^49^ As the human daily life consists mostly of activities in day light or under artificial light, the protection of cones is of primary importance and could suffice to retain most vital visual functions, such as visual acuity, even if the rods were partially lost.

In summary, our study provide confirmation that defective rod phototransduction by the *Gnat1* knock-out in mice increases their sensitivity to BLE damage, and therefore light exposure can be a risk factor for patients carrying *GNAT1* mutations. Patients with Oguchi disease could be at an even higher risk of these damaging light effects as the *Grk1^−/−^* and *Arr1^−/−^* mice were extremely sensitive to light damage. Interestingly, Oguchi disease patients, and congenital stationary night blindness patients in general, frequently experience a strong photophobia.^50^ Collectively, our results together with recent clinical findings^27^,^28^,^45^–^47^ suggest that the stationary nature of diseases caused by *GNAT1, SAG (Arr1),* and *GRK1* mutations should be taken with caution. We also report that the systems pharmacology–based therapy by coadministration of G_s_- (e.g., adrenergic β_1_-blockers) and Gq-coupled (e.g., adrenergic α_1_-blockers) receptor antagonists and G^i^-coupled receptor agonists (e.g., dopamine type-2 receptor agonists) may provide a potential disease-modifying treatment strategy for retinal degenerative diseases. In this report, we demonstrated its protective effects in *Gnat1^−/−^*, *Grk1^−/−^*, and *Arr1^−/−^* mouse models, which all carry defects in distinct parts of the rod phototransduction cascade. Future studies will focus on the investigation of systems pharmacology–based therapeutic effects and mechanisms in chronic disease models paving the way to clinical trials.

## Supplementary Material

Supplement 1
