# Supplementary material for: A Mixture of U.S. Food and Drug Administration–Approved Monoaminergic Drugs Protects the Retina From Light Damage in Diverse Models of Night Blindness
Source: Invest Ophthalmol Vis Sci. 2019 Apr;60(5):1442–53. doi: 10.1167/iovs.19-26560 (PMC6736410; doi:10.1167/iovs.19-26560)
Supplement: Supplement 1 [file iovs-60-04-64_s01.pdf]

SUPPLEMENTARY MATERIAL

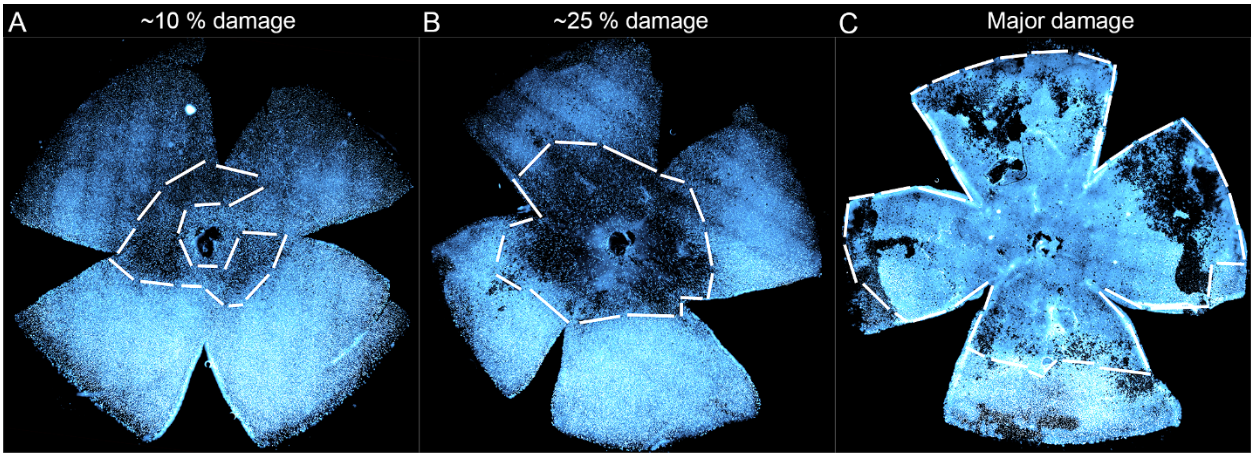

Figure S1. Illustrative sample of the variable extend of light-damage to cones in *Grk1*$^{-/-}$ mice (vehicle-treated group). Representative images of S-opsin stained flat-mounted retinas. (A) A mildly damaged retina. (B) A moderately damaged retina, which represents mean damage extend in vehicle-treated *Grk1*$^{-/-}$ mice. A subset of *Grk1*$^{-/-}$ retinas showed drastic damage to cone population as exemplified in panel C. Note that image is overexposed to highlight decreased cone density.
